# Decoding the phosphorylation landscape of ITPR3 across calcium signaling and regulatory networks in cancer

**DOI:** 10.3389/fbinf.2026.1972498

**Published:** 2026-10-21

**Authors:** Shanmitha B. Rai, Ayadathil Sujina, Sreeshma Ravindran Kammarambath, Althaf Mahin, Suhail Subair, Alimath Sambreena, S. Pavithra, Rajesh Raju, Akhina Palollathil

**Affiliations:** 1 Centre for Integrative Omics Data Science (CIODS), Yenepoya (Deemed to Be University), Mangalore, Karnataka, India; 2 School of Computer Science and Engineering, Vellore Institute of Technology, Chennai, Tamil Nadu, India

**Keywords:** Ca2+ signaling, endoplasmic reticulum, ITPR3, phosphoproteomics, proteoglycans

## Abstract

**Introduction:**

ITPR3 is an endoplasmic reticulum Ca^2+^ release channel that plays an important role in intracellular calcium signaling and has been implicated in cancer-associated cellular processes. Although several phosphorylation sites have been identified in ITPR3, the downstream regulatory networks associated with individual ITPR3 phosphosites remain poorly understood.

**Materials and Methods:**

In this study, we integratively analysed the publicly available human phosphoproteomic studies to characterize the phosphorylation landscape of ITPR3. Qualitative profiling and quantitative differential datasets were curated to identify phosphosites, followed by co-regulation analysis, functional enrichment, protein-protein interaction network analysis, upstream kinase identification and analysis of cancer-associated phosphoproteomic data using cProSite.

**Results and Discussion:**

Analysis of qualitative profiling and quantitative differential phosphoproteomic datasets identified four predominant ITPR3 phosphosites (S1832, S934, S2670, and S916) based on their recurrent detection and perturbation across independent datasets. Functional enrichment analysis revealed associations with chromatin organization, transcriptional regulation, RNA processing, cytoskeletal organization, and cellular signaling, indicating distinct regulatory patterns across ITPR3 phosphosites. Mapping of co-regulated proteins to the KEGG calcium signaling pathway identified multiple components involved in receptor-mediated signaling, IP_3_ generation, intracellular Ca^2+^ release, store-operated calcium entry, and calcium-responsive signaling. Furthermore, analysis of cancer-derived phosphoproteomic datasets showed the recurrent detection of the four predominant phosphosites across diverse cancer cell lines. Analysis using cProSite revealed distinct tumor-specific phosphorylation patterns, including significant differential phosphorylation of S1832 residue in pancreatic ductal adenocarcinoma, S2670 in breast cancer, S934 in ovarian cancer and S916 in kidney cancer. These findings provide a comprehensive view of ITPR3 phosphorylation and highlight potential regulatory networks connecting specific ITPR3 phosphosites with calcium signaling and cancer-associated processes. Further experimental studies are needed to determine the functional role of these phosphosites and to establish their association with ITPR3-mediated calcium signaling and tumor biology.

## 1 Introduction

Inositol 1,4,5-trisphosphate receptors (IP3Rs) are the intracellular calcium release channels predominantly localized to the endoplasmic reticulum, where they mediate the release of Ca^2+^ from intracellular stores in response to binding of inositol 1,4,5-trisphosphate (IP3), a second messenger generated downstream of G protein-coupled receptor (GPCR) and receptor tyrosine kinase signaling pathways (Gambardella et al., 2020; Ronkko et al., 2020). In mammals, the IP3R family comprises three isoforms, ITPR1 (IP3R1), ITPR2 (IP3R2), and ITPR3 (IP3R3), which share high structural similarity but exhibit distinct tissue distributions, regulatory properties, and sensitivities to IP3, ATP, and Ca^2+^ (Vervloessem et al., 2015).

IP3R3 is a tetrameric intracellular Ca^2+^ release channel with a molecular mass of approximately 304 kDa (UniProt, 2025) and is encoded by the ITPR3 gene located on chromosome 6p21.31. (Hornbeck et al., 2012; Sayers et al., 2025). Each subunit comprises an N-terminal IP3-binding region, a large cytoplasmic regulatory domain that serves as a platform for protein-protein interactions and post-translational modifications, and a C-terminal transmembrane pore responsible for Ca^2+^ conductance. Structurally, ITPR3 contains five conserved domains: the Ins145_P3_rec (Inositol 1,4,5-trisphosphate/ryanodine receptor), MIR (Mannosyltransferase Inositol 1,4,5- trisphosphate receptor Ryanodine receptor (RyR)), two RYDR_ITPR (Ryanodine Receptor and IP3 Receptor), RIH (Ryanodine Receptor/Inositol 1,4,5-trisphosphate Receptor Homology), and the ion transport domain. These domains collectively contribute to ligand recognition, channel regulation and ion transport (Ponting, 2000; Bosanac et al., 2005). By regulating intracellular Ca^2+^ dynamics, ITPR3 participates in diverse cellular processes, including proliferation, apoptosis, migration, gene transcription, and cellular homeostasis (Rodrigues et al., 2022). ITPR3 is broadly expressed in epithelial, secretory and immune cells in the pancreas, liver, gastrointestinal tract, salivary glands and lymphocytes (Gambardella et al., 2020). Dysregulated ITPR3 expression and Ca^2+^ signaling have been implicated in multiple pathological conditions, including cancer, where altered ITPR3 activity can influence cell survival and apoptotic responses. In cancer, ITPR3 knockdown has been reported to promote apoptosis, whereas its overexpression suppresses apoptosis (Akl and Bultynck, 2013). Furthermore, pathogenic variants in ITPR3 (c.4271C > T, c.7570C > G, c.7570C > T) have been associated with intermediate Charcot-Marie-Tooth disease and combined immunodeficiency, highlighting the importance of ITPR3-mediated Ca^2+^ signaling in human health and disease (Cabello-Murgui et al., 2024; Yu et al., 2025). Several phosphorylation sites have been identified within the regulatory region of ITPR3, including S916, S934, and S1832. However, experimental studies suggest that their modification does not directly alter IP3R3-mediated Ca^2+^ release, indicating that they may participate in alternative regulatory mechanisms such as protein-protein interactions or signaling complex assembly (Soulsby and Wojcikiewicz, 2007). To investigate the possible phosphoregulatory landscape of ITPR3, we conducted an integrative analysis of publicly available phosphoproteomics datasets. In addition to upstream kinases and interaction networks, we identified the most common phosphorylation sites and examined their co-regulation patterns. These observations may provide initial insights into site-specific regulatory mechanisms of ITPR3 and could serve as a foundation for future functional studies.

## 2 Materials and Methods

### 2.1 Curation of ITPR3 phosphosites from public phosphoproteomic studies

To systematically characterize phosphosite-specific regulation associated with ITPR3, publicly available human cellular phosphoproteomic studies were identified through a comprehensive literature survey. A PubMed search was performed using the query terms “phosphoproteomics” OR “phosphoproteome”, with the search conducted up to May 2024. Publications categorized as “Plant” or “Review” were excluded from the search results. The search retrieved approximately 3,900 articles, which were subsequently screened to identify high-throughput studies reporting human phosphoproteomic data containing ITPR3 phosphosites, resulting in 160 publications. Details on the quantification approaches (label-free, TMT, and SILAC), mass spectrometry instruments (e.g., Orbitrap), phosphopeptide enrichment methods (TiO_2_ and Fe-IMAC), and Class I site identification criteria are provided in Supplementary Tables S1 and S2.

Subsequently, data curation and analysis of ITPR3 phosphoproteomic datasets were performed using a methodology previously established by our team (Fathima et al., 2025; Sujina et al., 2025; Thomas et al., 2025; Rai et al., 2026; Shaji et al., 2026). Based on the experimental design, the datasets were categorized as qualitative profile datasets (test conditions and control as independent phosphosite profile datasets) and quantitative differential datasets (directly compared test biological or experimental conditions against their corresponding controls).

The curated datasets encompassed diverse experimental conditions and mass spectrometry-based quantification strategies, including both label-based and label-free quantification approaches. The fold-change thresholds were applied within each individual quantitative dataset to classify significantly regulated phosphosites, rather than directly comparing fold-change values across different quantification strategies. To obtain high-confidence phosphosite identifications and minimize potential false-positive assignments, only phosphosites with a localization probability of ≥75% or an A-score >13 were considered. To further enhance biological relevance and statistical robustness, differential datasets were filtered using a significance threshold of p < 0.05, along with fold-change cutoffs of ≥1.3 for upregulated sites and ≤0.76 for downregulated sites.

The datasets were further classified according to the enriched phosphorylated amino acid residue, namely, serine (Ser), threonine (Thr), or tyrosine (Tyr). Identified phosphosites were mapped to their corresponding UniProt accessions using an in-house phosphosite mapping pipeline (UniProt release on 13 May 2023) (UniProt, 2023), followed by gene symbol standardization according to HGNC annotations (Tweedie et al., 2021). The resulting curated and harmonized datasets were subsequently used for differential regulation assessment, phosphosite frequency analysis, downstream co-regulation and interaction analyses. The overall workflow used in this study is outlined in Figure 1.

**FIGURE 1:**
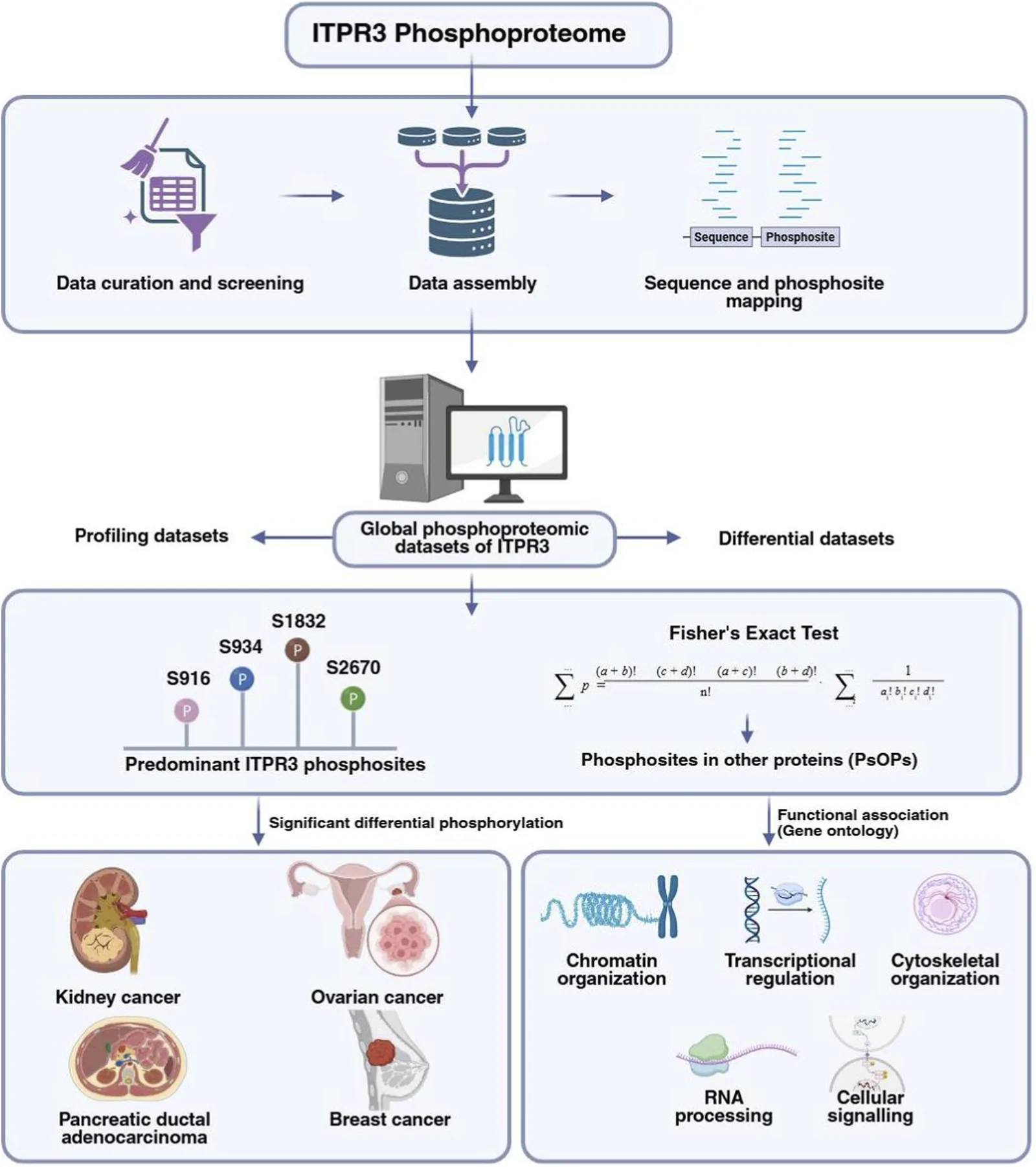
Workflow of the data assembly and analysis of ITPR3 phosphosites and their co-regulated phosphosites in other proteins. Publicly available phosphoproteomic datasets were curated, assembled, and mapped. Predominant ITPR3 phosphosites were identified, followed by Fisher’s Exact Test-based co-regulation analysis to identify high-confidence positively and negatively co-regulated PsOPs. The resulting co-regulated phosphosites were further analyzed for kinase-substrate relationships, cancer-associated phosphorylation patterns and functional associations. Figure created using bioRender https://www.biorender.com/.

### 2.2 Identification of predominant ITPR3 phosphosites

To determine the predominant phosphorylation sites of ITPR3, human cellular global phosphoproteomics datasets enriched for serine (S), threonine (T), and tyrosine (Y) phosphopeptides were systematically examined. High-confidence Class-1 ITPR3 phosphosites defined by a localization probability ≥75% or A-score ≥13, were retrieved from the curated qualitative profile and quantitative differential phosphoproteomics datasets. The occurrence of each phosphosite was assessed based on the number of qualitative profile datasets in which the site was detected and the number of quantitative datasets in which it showed significant differential regulation. Based on these frequencies, phosphosites were ranked to prioritize consistently observed phosphorylation events across independent studies. Phosphosites identified most frequently in both qualitative profiling and quantitative differential datasets were designated as predominant ITPR3 phosphosites. Phosphorylation sites reported exclusively through targeted approaches, such as phospho-specific antibody-based assays or mutagenesis studies, were not consistently identified as Class-1 phosphosites across large-scale phosphoproteomics datasets and were therefore not included in the subsequent classification. The distribution and occurrence of ITPR3 phosphosites along the protein sequence were visualized using lollipop plots generated with the R/Bioconductor package trackViewer, enabling representation of the spatial distribution and frequency of phosphorylation events across the protein.

### 2.3 Identification of similarly and oppositely co-regulated phosphosites predominant to ITPR3 phosphosites

Quantitative human cellular phosphoproteomic datasets were further analyzed to identify phosphosites in other proteins (PsOPs) that were coordinately regulated with the predominant ITPR3 phosphosites (S1832, S934, S2670, S916). Analyses were performed separately for each predominant ITPR3 phosphosite to assess site-specific regulatory patterns. Due to differences in experimental conditions, biological systems, and analytical platforms among the published studies, raw data re-analysis was not feasible. Therefore, the curated quantitative differential datasets were used irrespective of potential overrepresentation of specific experimental contexts. For each predominant ITPR3 phosphosite, phosphorylation events were categorized as upregulated (U) or downregulated (D). Based on the regulation status of the corresponding PsOPs, four possible combinations were obtained: UU, UD, DU, and DD. PsOPs that were upregulated or downregulated concordantly with ITPR3 phosphosites (UU and DD) were considered candidates for positive co-regulation, whereas PsOPs showing opposing regulation (UD and DU) were considered candidates for negative co-regulation. Accordingly, phosphosite pairs were grouped into UUDD for positive co-regulation and UDDU for negative co-regulation.

A one-sided Fisher’s exact test (FET) was used to assess the significance of co-regulation based on the co-occurrence of phosphosite regulation across quantitative differential datasets (Kammarambath et al., 2025; Pai et al., 2026; Palollathil et al., 2026; Poojari et al., 2026). PsOP pairs with a FET p-value <0.05 in either the UUDD or UDDU category were considered statistically significant.

$$\sum p=\frac{\left(a+b\right)!\left(c+d\right)!\left(a+c\right)!\left(b+d\right)!}{n!}\;\underset{i}{\sum }\frac{1}{a_{i}!b_{i}!c_{i}!d_{i}!}$$

In the contingency table: ‘***a***’ n_00 represents the number of experimental conditions in which both phosphosites were not differentially regulated. ‘***b***’ (n_U0+n_D0+n_0U + n_0D) represents the number of conditions where either of the phosphosite was differentially regulated (irrespective of upregulated or downregulated). ‘***c***’ (n_UD + n_DU) corresponds to the number of experimental conditions showing opposite co-regulation between two phosphosites. ‘***d***’ (nUU + nDD) represents the number of conditions in which both phosphosites exhibited identical co-regulation (both were either upregulated or downregulated).

To reduce potential bias arising from repeated experimental conditions and overrepresentation of particular studies or stimuli, additional filtering criteria were applied. For the co-regulation analysis, PsOPs associated with the predominant ITPR3 phosphosites detected in at least 10% of the total datasets were considered for downstream analysis. Additionally, to ensure biological robustness, PsOPs associated with each ITPR3 phosphosite were required to be detected in at least three independent studies, represented by distinct PubMed IDs and experimental conditions. These criteria helped minimize dataset-specific bias and retain phosphosite co-regulatory associations that were biologically meaningful and reproducible. The resulting high-confidence positively and negatively co-regulated PsOPs for each predominant ITPR3 phosphosite were subsequently used for downstream bioinformatic analyses, including functional enrichment, protein-protein interaction analysis, and kinase-substrate relationship prediction.

### 2.4 Identification of ITPR3 interacting proteins

To characterize the ITPR3 interaction network, experimentally supported and predicted protein interactors were collected from multiple curated protein-protein interaction databases. Binary and complex interactors associated with ITPR3 were obtained from the Human Protein Reference Database (HPRD) (Keshava Prasad et al., 2009), Biomolecular Interaction Network Database (BIND) (Bader et al., 2003), BioGRID (Oughtred et al., 2021), ConsensusPathDB release 35 (Kamburov and Herwig, 2022), NetPath (Kandasamy et al., 2010), CORUM (Tsitsiridis et al., 2023), and RegPhos 2.0 (Huang et al., 2014). Datasets from these resources were combined to generate a comprehensive map of proteins associated with ITPR3.

### 2.5 Prediction and identification of upstream kinases of ITPR3 phosphosites

Potential upstream kinases associated with ITPR3 phosphorylation sites were identified using a combination of computational prediction tools and experimentally derived kinase-substrate resources. Kinase prediction and annotation were performed using complementary computational and experimental resources. Kinase associations for individual ITPR3 phosphosites were obtained from NetworKIN (accessed 4 January 2023) (Linding et al., 2008) and AKID (accessed 24 May 2023) (Parca et al., 2019). Experimentally derived kinase-substrate relationships were obtained from iKiP-DB (Mari et al., 2022) and PhosphoSitePlus (downloaded on 22 May 2023) (Hornbeck et al., 2012). In addition, kinase associations reported by Johnson et al. (2023) based on synthetic peptide screening across the human kinome were incorporated, using a 90th-percentile confidence cutoff (Johnson et al., 2023).

### 2.6 cProSite-based cancer phosphoproteomics analysis

The Cancer Proteogenomic Data Analysis site (cProSite) (Wang et al., 2023) is an interactive web-based resource for exploring and visualizing large-scale proteomic and phosphoproteomic datasets. To assess the expression patterns of ITPR3 phosphosites across cancer types, cProSite (https://cprosite.ccr.cancer.gov/) was used.

### 2.7 Functional enrichment analysis and data visualization

Functional gene enrichment analysis of proteins positively and negatively co-regulated with ITPR3 was performed using the Enrichr tool (Xie et al., 2021). Multiple sequence alignment (MSA) was performed using Clustal Omega (Sievers and Higgins, 2018), Network construction and visualization were carried out using Cytoscape (Shannon et al., 2003), ClueGO (Bindea et al., 2009), and cytoHubba (Chin et al., 2014) and RAWGraphs 2.0 (Mauri et al., 2017) (https://app.rawgraphs.io/) was used to create a dendrogram. KEGG database was used to visualize the Calcium signaling pathway (Kanehisa et al., 2023).

## 3 Results and Discussion

### 3.1 Assembly and characterization of the ITPR3 phosphoproteome

We screened publicly available human phosphoproteomics studies indexed in PubMed to identify studies reporting ITPR3 phosphorylation. The resulting articles were subjected to data curation to fetch datasets on ITPR3. These included 789 qualitative profile datasets, in which phosphosites were identified and catalogued across diverse biological conditions, and 187 quantitative differential datasets, in which phosphosite abundance was compared between experimental conditions and their corresponding controls. Mapping of phosphosites with ITPR3 identified 24 phosphosites from profiling datasets and 18 phosphosites from differential expression datasets. Across the profiling datasets, S1832 (frequency = 655), S2670 (frequency = 644), S934 (frequency = 561), and S916 (frequency = 215) were the most frequently identified phosphosites. A similar pattern was observed in the differential datasets, with S1832, S934, S2670, and S916 showing the highest detection frequencies of 99, 74, 69, and 24, respectively (Supplementary Tables S1 and S2). These sites were therefore considered as the predominant phosphosites of ITPR3 based on their high frequency of occurrence and were prioritized for further analyses. Among the predominant phosphorylation sites identified in ITPR3, S916 and S934 are located in the inter-domain region between the two RYDR domains. S1832 is positioned between the second RYDR and the RIH domain, whereas S2670 is located toward the C-terminal end of the protein. Notably, all four predominant phosphosites are situated outside the IP_3_-binding and transmembrane channel domains (Figure 2).

**FIGURE 2:**
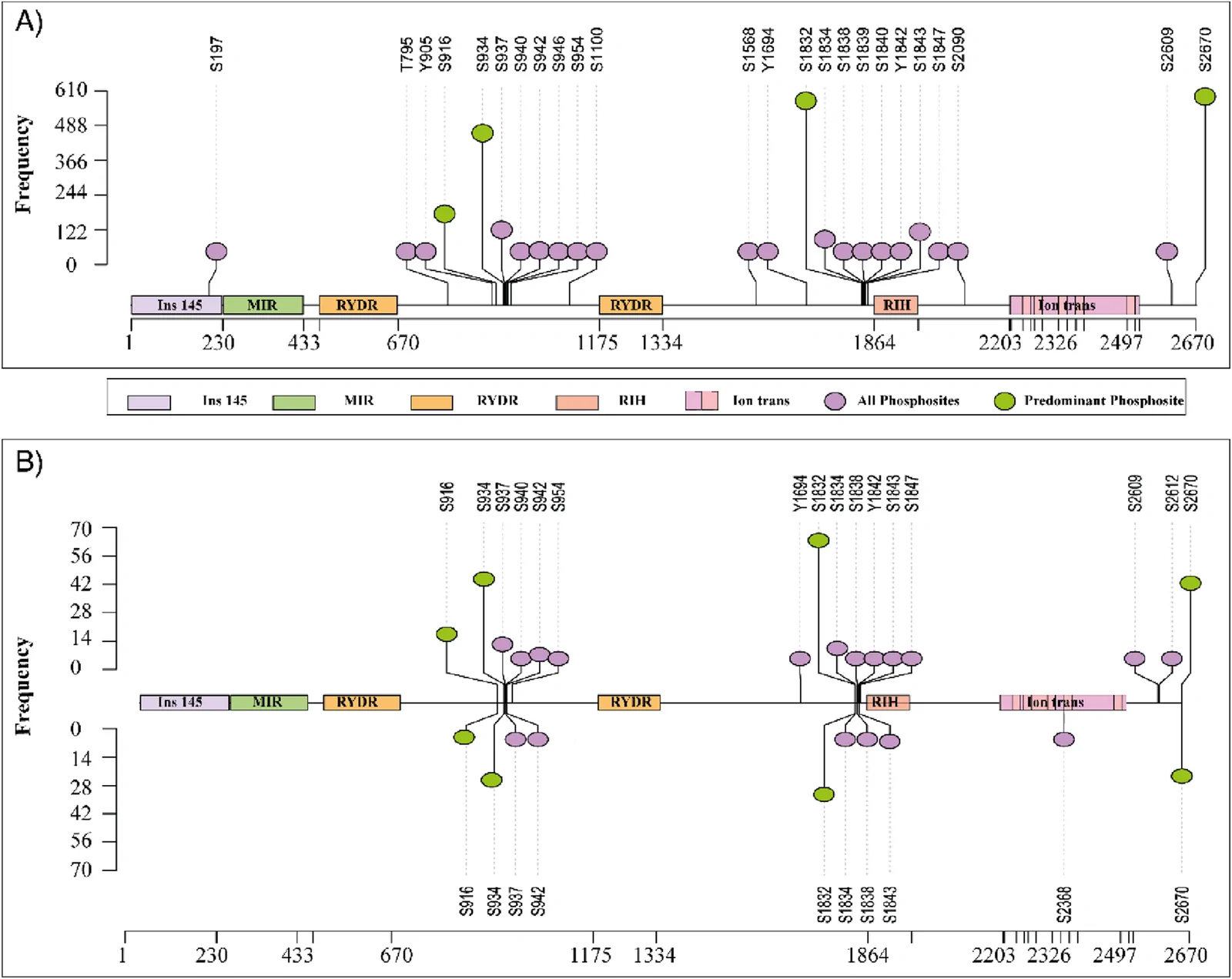
Lollipop plot depicting the phosphosite distribution of ITPR3 **(A)** Class-1 phosphosites identified in ITPR3 across qualitative profiling datasets. **(B)** Class-1 phosphosites identified in ITPR3 across quantitative differential datasets. Phosphosites plotted above the x-axis represent positively co-regulated phosphosites, whereas those below the x-axis represent negatively co-regulated phosphosites. The y-axis indicates the frequency of phosphosite co-regulation across datasets.

To evaluate the conservation of the predominant ITPR3 phosphosites within the IP_3_ receptor family, multiple sequence alignment of ITPR1, ITPR2, and ITPR3 was performed using Clustal Omega. The analysis revealed that the predominant phosphosites of ITPR3 was not conserved among the other IP_3_ receptor isoforms. To assess conservation across species, orthologous sequence analysis was performed using ITPR3 protein sequences from human, mouse, rat and bovine. In contrast to the inter-isoform comparison, all four predominant phosphosites (S1832, S934, S2670, and S916) were highly conserved across the species examined, indicating strong evolutionary preservation of these phosphosites and suggesting their potential functional importance.

### 3.2 Co-occurrence of ITPR3 phosphosites

Following the identification of the predominant ITPR3 phosphosites, we investigated their co-occurrence patterns. The co-occurrence of phosphosites is frequently associated with coordinated signaling events and may reflect a functional relationship between phosphosites (Li et al., 2017). To investigate the phosphorylation dynamics of ITPR3, we examined the co-regulation patterns of differentially regulated phosphosites across the assembled phosphoproteomics datasets. Among the identified phosphosites, S1832 emerged as a major co-regulated site, displaying strong positive associations with S2670, S934, and S916 with 27, 19, and 15 datasets, respectively. Notably, the strongest positive co-regulatory relationships were observed between S1832 and S2670 with co-regulation across 27 datasets, followed by S934 and S916, which showed positive co-regulation across 11 datasets, indicating frequent coordinated regulation of these phosphosites across multiple biological and experimental conditions. In contrast, negative co-regulation events were relatively infrequent and occurred at substantially lower frequencies (Figure 3).

**FIGURE 3:**
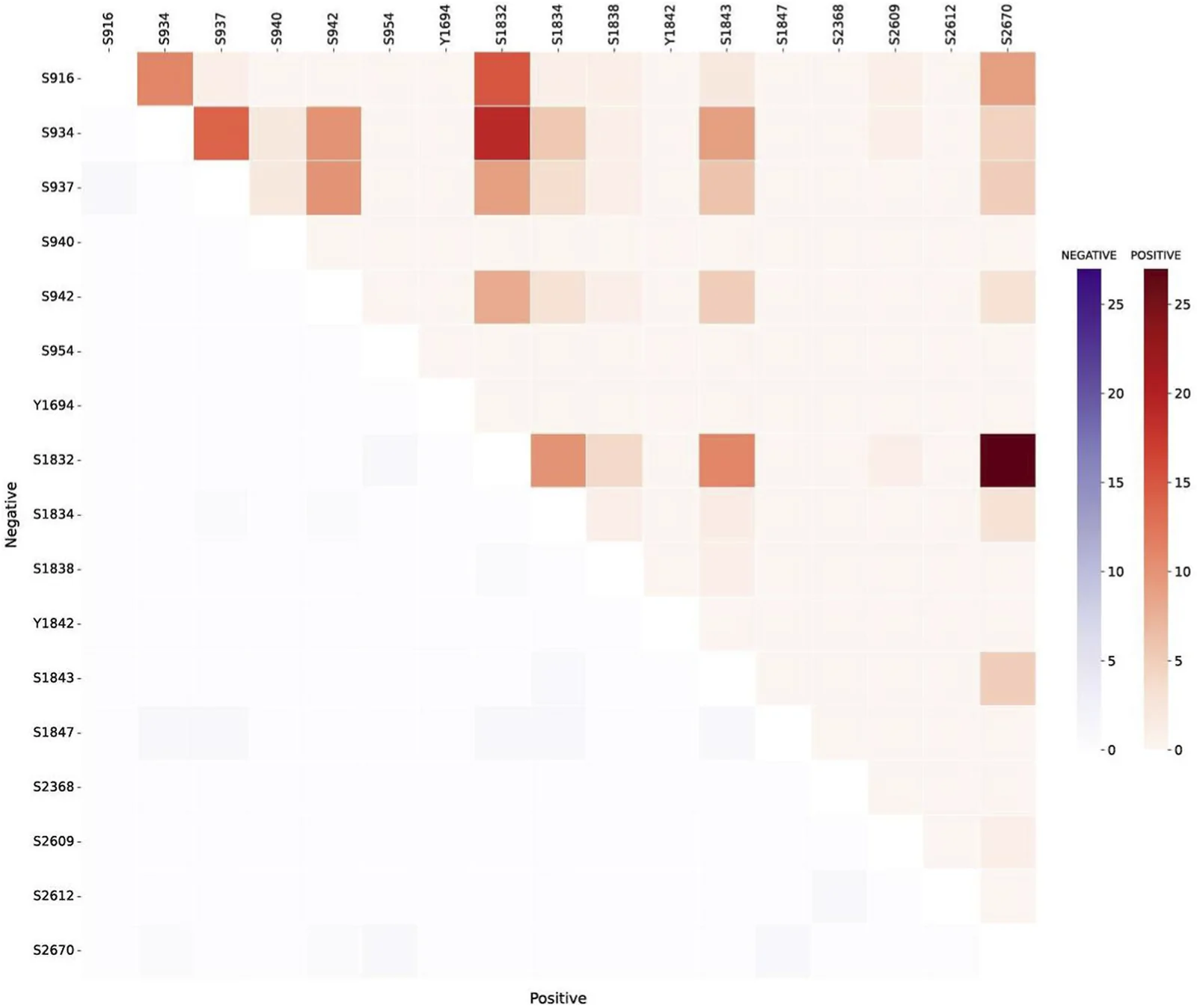
Heat map showing positive and negative co-occurrence patterns among ITPR3 phosphosites. Each cell represents the degree of co-occurrence between two sites within the ITPR3. Red indicates positive co-regulation, and Violet indicates negative co-regulation. Color intensity corresponds to the strength of the association, with darker shades representing higher co-occurrence.

### 3.3 Co-regulation analysis of predominant ITPR3 phosphosites

A systematic co-regulation analysis was conducted to assess the regulatory associations of the predominant ITPR3 PsOPs across independent phosphoproteomic datasets. Co-regulated PsOPs were identified based on the predefined statistical and reproducibility criteria described in the Materials and Methods section. Following application of these criteria, S1832 exhibited positive and negative co-regulation with 165 and 88 PsOPs, respectively (Supplementary Tables S3 and S4). S934 was positively co-regulated with 241 PsOPs and negatively co-regulated with 108 PsOPs (Supplementary Tables S5 and S6), whereas S2670 showed the largest number of positively co-regulated proteins, comprising 412 PsOPs and 61 negatively co-regulated PsOPs (Supplementary Tables S7 and S8). In contrast, S916 displayed the most extensive set of negatively co-regulated PsOPs, with 581 negatively co-regulated PsOPs and 339 positively co-regulated PsOPs (Supplementary Tables S9 and S10).

Among the top positively co-regulated PsOPs associated with ITPR3 (S1832) were ITPR3 (S2670), Ubiquitin-Specific Peptidase 10 (USP10) (S211), and Small, 11-kDa calcium-binding protein belonging to the S100 protein family (S100A13) (S32), whereas EH domain-containing protein 2 (EHD2) (S468), Chromosome X Open Reading Frame 38 (CXorf38) (T314), and Zinc finger CCCH-type containing 11 A (ZC3H11 A) (T3210) represented the strongest negatively co-regulated PsOPs (Figure 4A). For S934, the top positively co-regulated PsOPs included ITPR3 (S1832), Protein Phosphatase 1 Regulatory Subunit 12C (PPP1R12C) (S452), and Adherens junction formation factor (AFDN) (S1083), while Cell Division Cycle-Associated Protein 3 (CDCA3) (S29), Heterogeneous Nuclear Ribonucleoprotein M (HNRNPM) (S528), and Treacle ribosome biogenesis factor 1 (TCOF1) (S1376) showed the strongest negative associations (Figure 4B). S2670 was positively co-regulated with ITPR3 (S1832), RNA-Binding Motif 6 (RBM6) (S362), and Zinc finger protein 316 (ZNF316) (S10), whereas Golgi-specific brefeldin A-resistance guanine nucleotide exchange factor 1 (GBF1) (S1781), Microtubule-associated protein 1B (MAP1B) (T1788), and RING Finger Protein 214 (RNF214) (S47) were among the top negatively co-regulated PsOPs (Figure 4C). For S916, ITPR3 (S1832), ITPR3 (S934), and heterogeneous nuclear ribonucleoprotein U (HNRNPU) (S59) were the most strongly positively co-regulated PsOPs, while GRB10-interacting GYF protein 2 (GIGYF2) (S26), CD2-associated protein (CD2AP) (S514), and Eukaryotic translation initiation factor 4E-binding protein 1 (EIF4EBP1) (S65) exhibited the strongest negative co-regulatory associations (Figure 4D).

**FIGURE 4:**
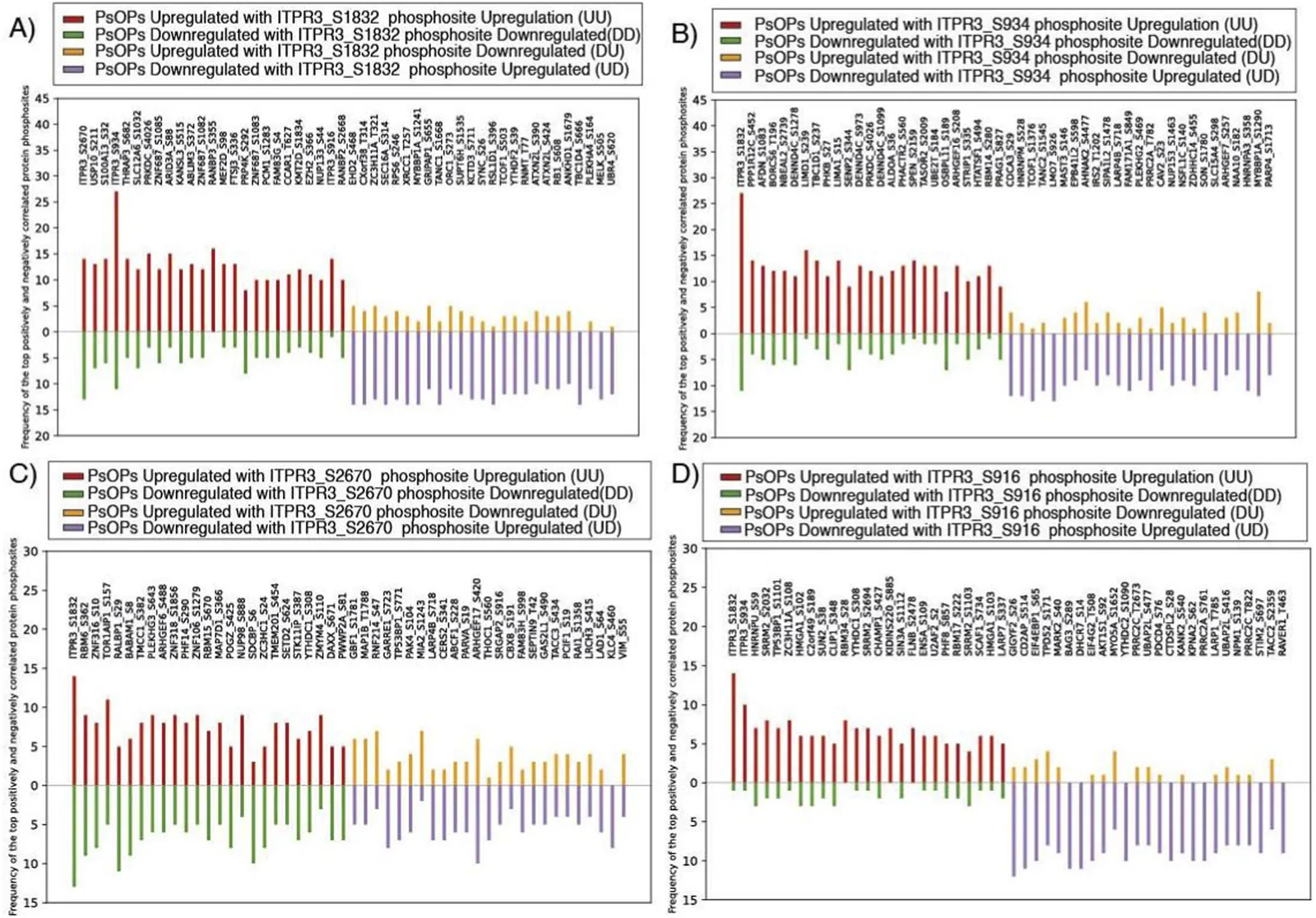
Bar plots summarize the 25 phosphosites of other proteins (PsOPs) showing the highest frequencies of positive and negative co-regulation with **(A)** S1832, **(B)** S934, **(C)** S2670, and **(D)** S916. Bars above and below the baseline represent positive and negative co-regulation, respectively, with colors distinguishing phosphosite upregulation and downregulation relative to the corresponding ITPR3 phosphosite.

Notably, several of the strongest positively co-regulated PsOPs corresponded to other ITPR3 phosphosites. S1832 showed strong positive co-regulation with S2670, while S934 and S916 were also strongly associated with S1832. In addition (S100A13) (S32), identified among the top positively co-regulated PsOPs associated with S1832, belongs to the S100 family of calcium-binding proteins (Al-Mutairy et al., 2023; Xia et al., 2025).

### 3.4 Biological processes associated with predominant ITPR3 phosphosites

Gene Ontology (GO) enrichment analysis was conducted using Enrichr to characterize the biological processes associated with the predominant ITPR3 phosphosites and their co-regulated PsOPs. Positively and negatively co-regulated proteins were analyzed separately (Xie et al., 2021). Positively co-regulated proteins associated with ITPR3 S1832 were significantly enriched in processes related to positive regulation of DNA-templated transcription, positive regulation of RNA biosynthetic processes, positive regulation of transcription by RNA polymerase II, chromatin organization, and heterochromatin formation. Proteins positively co-regulated with S2670 were enriched in chromatin organization, positive regulation of DNA-templated transcription, chromatin remodeling, positive regulation of transcription by RNA polymerase II, and negative regulation of the G2/M transition of the mitotic cell cycle. For S916, positively co-regulated proteins were primarily associated with RNA processing, mRNA processing, mRNA splicing via the spliceosome, regulation of mRNA splicing via the spliceosome, and chromatin organization. In contrast, proteins positively co-regulated with S934 were enriched in regulation of protein localization to the plasma membrane, regulation of small GTPase-mediated signal transduction, regulation of lamellipodium organization, protein localization to sites of DNA double-strand breaks, and cortical microtubule organization (Figure 5A).

**FIGURE 5:**
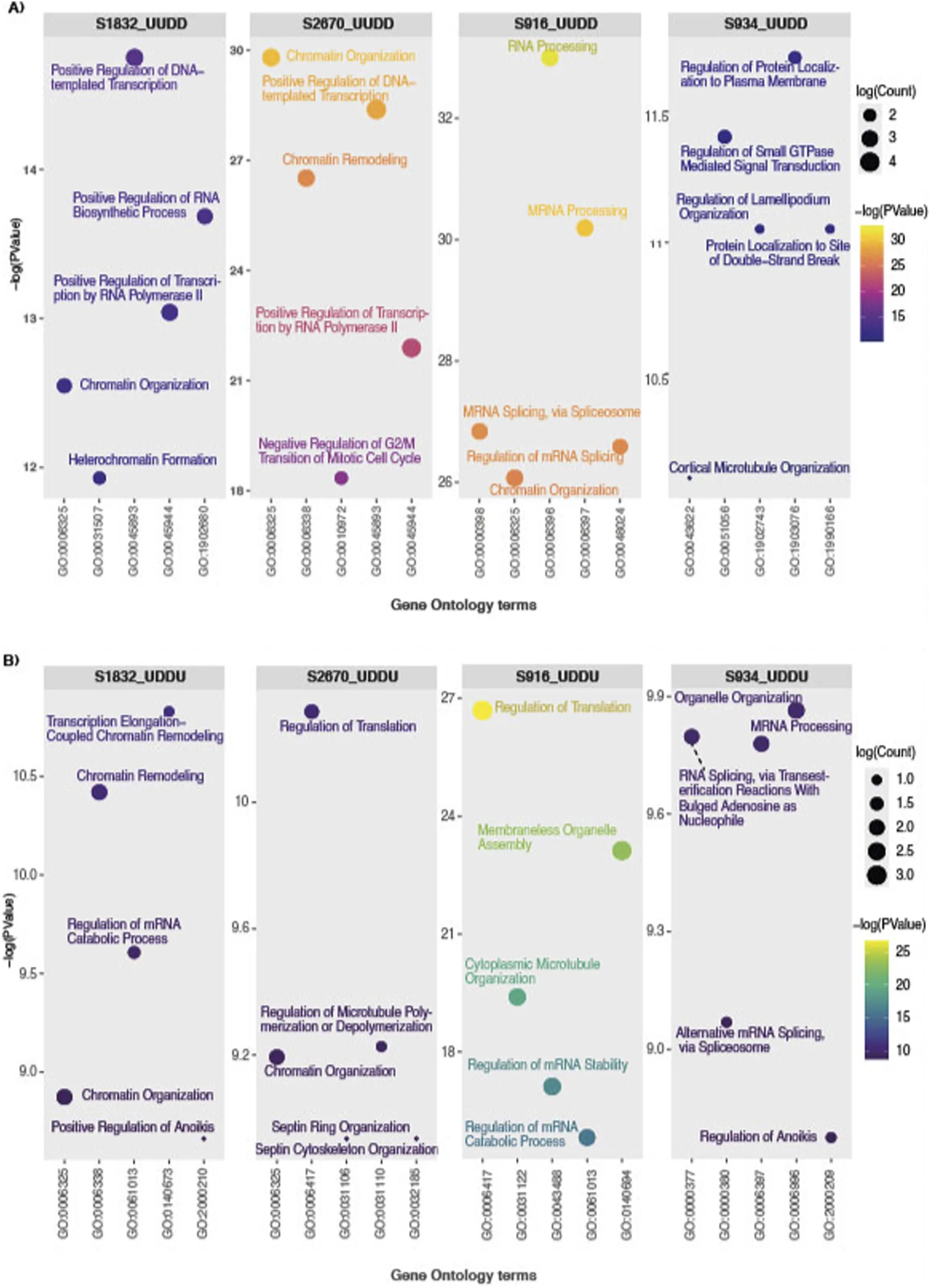
**(A)** Top enriched biological processes associated with proteins positively co-regulated (UUDD) with S1832, S2670, S916, and S934. **(B)** Top enriched biological processes associated with proteins negatively co-regulated (UDDU) with the corresponding phosphosites. Bubble size represents gene count, while color indicates enrichment significance (−log10 p-value).

Analysis of negatively co-regulated proteins revealed distinct functional enrichments for each phosphosite. Proteins negatively co-regulated with S1832 were enriched in transcription elongation-coupled chromatin remodeling, chromatin remodeling, regulation of mRNA catabolic processes, chromatin organization, and heterochromatin formation. Negatively co-regulated proteins associated with S2670 were enriched in the regulation of translation, regulation of microtubule polymerization or depolymerization, chromatin organization, septin cytoskeleton organization, and septin ring organization. For S916, negatively co-regulated proteins were enriched in regulation of translation, membraneless organelle assembly, cytoplasmic microtubule organization, regulation of mRNA stability, and regulation of mRNA catabolic processes. Proteins negatively co-regulated with S934 were associated with organelle organization, RNA splicing via transesterification reactions with bulged adenosine as nucleophile, mRNA processing, alternative mRNA splicing via the spliceosome, and regulation of anoikis. Together, these findings suggest that distinct ITPR3 phosphosites are associated with different regulatory programs involving transcriptional control, chromatin dynamics, RNA processing, cytoskeletal organization, and cellular signaling pathways (Figure 5B).

To further identify the central regulatory components within the co-regulated protein networks, hub gene analysis was performed using the cytoHubba plugin in Cytoscape, which identifies highly connected nodes within biological interaction networks (Chin et al., 2014). The top 10 hub genes were selected based on the shortest-path ranking method, for each predominant ITPR3 phosphosite. Overall, the identified hub genes were involved in the top-enriched biological processes identified by Gene Ontology enrichment analysis. For S1832, both positively and negatively co-regulated hub genes were predominantly associated with chromatin organization, chromatin remodeling, transcriptional regulation, and DNA damage response, consistent with the enriched GO terms. Likewise, the hub genes associated with S916 largely reflected the enrichment of RNA processing, mRNA splicing, translation, and cytoskeletal organization. In the case of S2670 and S934, several hub genes corresponded to the major enriched biological processes, indicating that these phosphosites may participate in multiple biological pathways. Collectively, these findings provide complementary support for the functional enrichment analysis and highlight the central regulatory proteins associated with the predominant ITPR3 phosphosites (Supplementary Figure S2).

### 3.5 Upstream kinase co-regulation associated with predominant ITPR3 phosphosites

Potential upstream kinases associated with the predominant ITPR3 phosphosites were identified using computational prediction approaches, including NetworKIN/AKID and the sequence-based specificity analysis described by Johnson et al. (2023) to infer the potential upstream kinase responsible for ITPR3 phosphorylation. Overall, 20 unique kinases were identified among the PsOPs co-regulated with predominant phosphosites of ITPR3 based on the kinase-substrate relationship reported by Johnson et al. (2023), while six kinases were predicted using NetworKIN/AKID. The positively or negatively co-regulated kinases associated with predominant ITPR3 phosphosites are represented in Figure 6.

**FIGURE 6:**
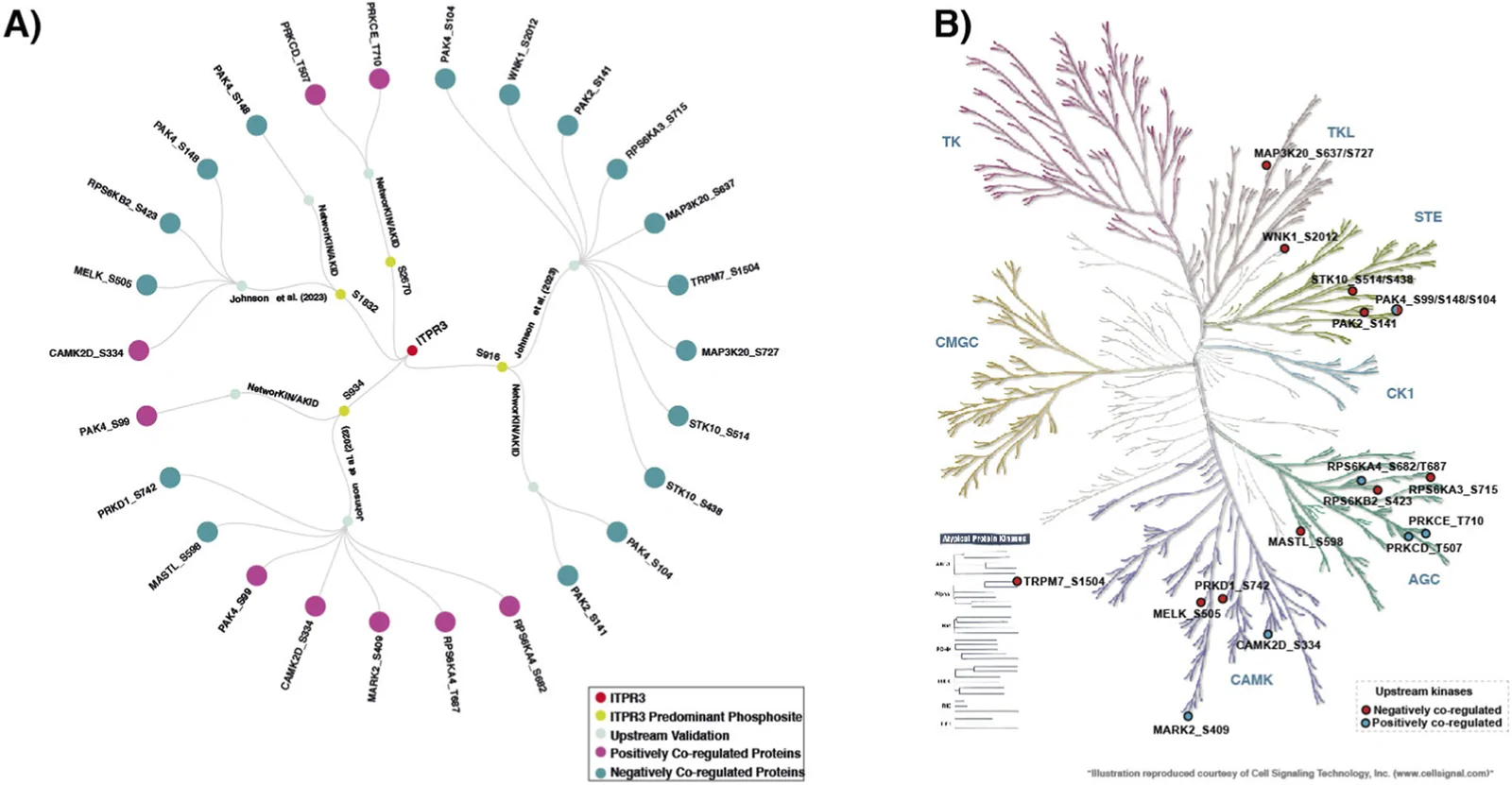
Upstream kinase network and KinMap classification of ITPR3 regulatory kinases. **(A)** The network diagram illustrates the upstream kinases of ITPR3 and their associated phosphosites. **(B)** KinMap showing the phylogenetic distribution of ITPR3 -associated upstream kinases.

Among the Johnson et al. predicted kinases, Calcium/calmodulin-dependent protein kinase II delta (CAMK2D) (S334) exhibited positive co-regulation with predominant site S1832, while Maternal embryonic leucine zipper kinase (MELK) (S50), ribosomal protein S6 kinase beta-2 RPS6KB2 (S423), and p21-activated kinase 4 (PAK4) (S148) showed negative co-regulation. Additionally, PAK4 (S148), negatively co-regulated with S1832, was also predicted as an upstream kinase using NetworKIN/AKID. For the predominant phosphosite S934, ribosomal protein S6 kinase A4 (RPS6KA4) (T687, S682), microtubule affinity-regulating kinase 2 (MARK2 S409), and calcium/calmodulin-dependent protein kinase II delta (CAMK2D) (S334) exhibited positive co-regulation and were identified from the analysis of Johnson et al. (2023). In addition, p21-activated kinase 4 (PAK4) (S99) was reported as a positively co-regulated upstream kinase by both Johnson et al. (2023) and NetworKIN/AKID. In contrast, microtubule-associated serine/threonine kinase-like (MASTL) (S598) and protein kinase D1 (PRKD1) (S742) exhibited negative co-regulation and were identified as potential upstream regulators by Johnson et al. (2023).

For the predominant phosphosite S2670, positively co-regulated kinases, such as protein kinase C delta (PRKCD) (T507) and protein kinase C epsilon (PRKCE) (T710), were identified as potential upstream kinases using NetworKIN/AKID. Among the predominant phosphosites, S916 exhibited the most extensive kinase regulatory network. WNK lysine-deficient protein kinase 1 (WNK1) (S2012), Ribosomal protein S6 kinase alpha-3 (RPS6KA3) (S715), Mitogen-activated protein kinase kinase 20 (MAP3K20) (S637 and S727), Transient receptor potential cation channel subfamily M member 7 (TRPM7) (S1504), Serine/threonine kinase 10 (STK10) (S438 and S514), and p21 (RAC1) activated kinase 2 (PAK2) (S143) exhibited negative co-regulation and were identified from the analysis of Johnson et al. (2023). In addition, PAK4 (S104) and PAK2 (S141) were identified by both Johnson et al. (2023) and NetworKIN/AKID as negatively co-regulated upstream kinases (Figure 6A; Supplementary Table S11). The kinome map highlights the phylogenetic distribution of upstream kinases that are positively and negatively co-regulated with ITPR3. The positively co-regulated upstream kinases were mainly categorized under the AGC kinase group (PRKCD, PRKCE, RPS6KA4), followed by the CAMK (CAMK2D, MARK2) and STE (PAK4) kinase group. The negatively co-regulated kinases were predominantly distributed across the AGC (MASTL, RPS6KB2, RPS6KA3) and STE (PAK4, PAK2, STK10) kinase groups, with three kinases in each group. The CAMK group comprised two kinases (MELK, PRKD1), while the Atypical (TRPM7), Other (WNK1), and TKL (MAP3K20) kinase groups each contained one kinase (Figure 6B).

### 3.6 Analysis of co-regulated phosphosites in ITPR3 binary and complex interactors

To explore the potential functional interactions associated with ITPR3 phosphorylation, we examined the protein interaction network of proteins co-regulated with the predominant ITPR3 phosphosites. For S1832, five binary interactors and 35 complex interactors were observed among positively co-regulated PsOPs, while one binary and 10 complex interactors were identified within negatively co-regulated PsOPs. In the case of S934, nine binary and 45 complex interactors showed positive co-regulation, whereas two binary and 20 complex interactors showed negative co-regulation. For S2670, two binary and 46 complex interactors were positively co-regulated, whereas one binary and 14 complex interactors were negatively co-regulated. For S916, five binary and 58 complex interactors were positively co-regulated, whereas six binary and 122 complex interactors were negatively co-regulated (Figures 7, 8A; Supplementary Tables S12 and S13).

**FIGURE 7:**
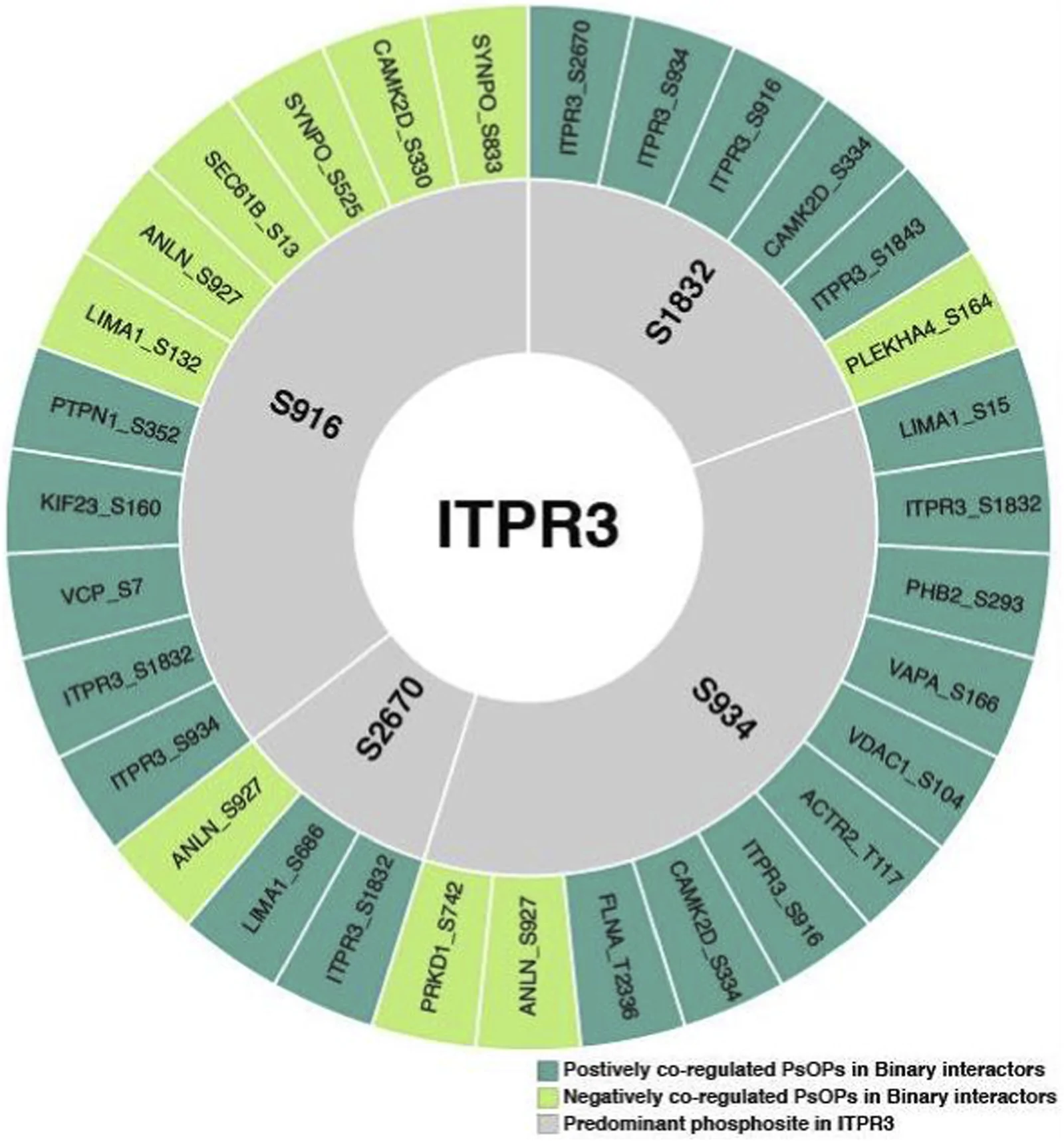
Protein-protein interaction network of ITPR3 and its binary interactors. The interaction network illustrates the positive and negative co-regulation of ITPR3 predominant phosphosites with phosphosites in binary interactors.

**FIGURE 8:**
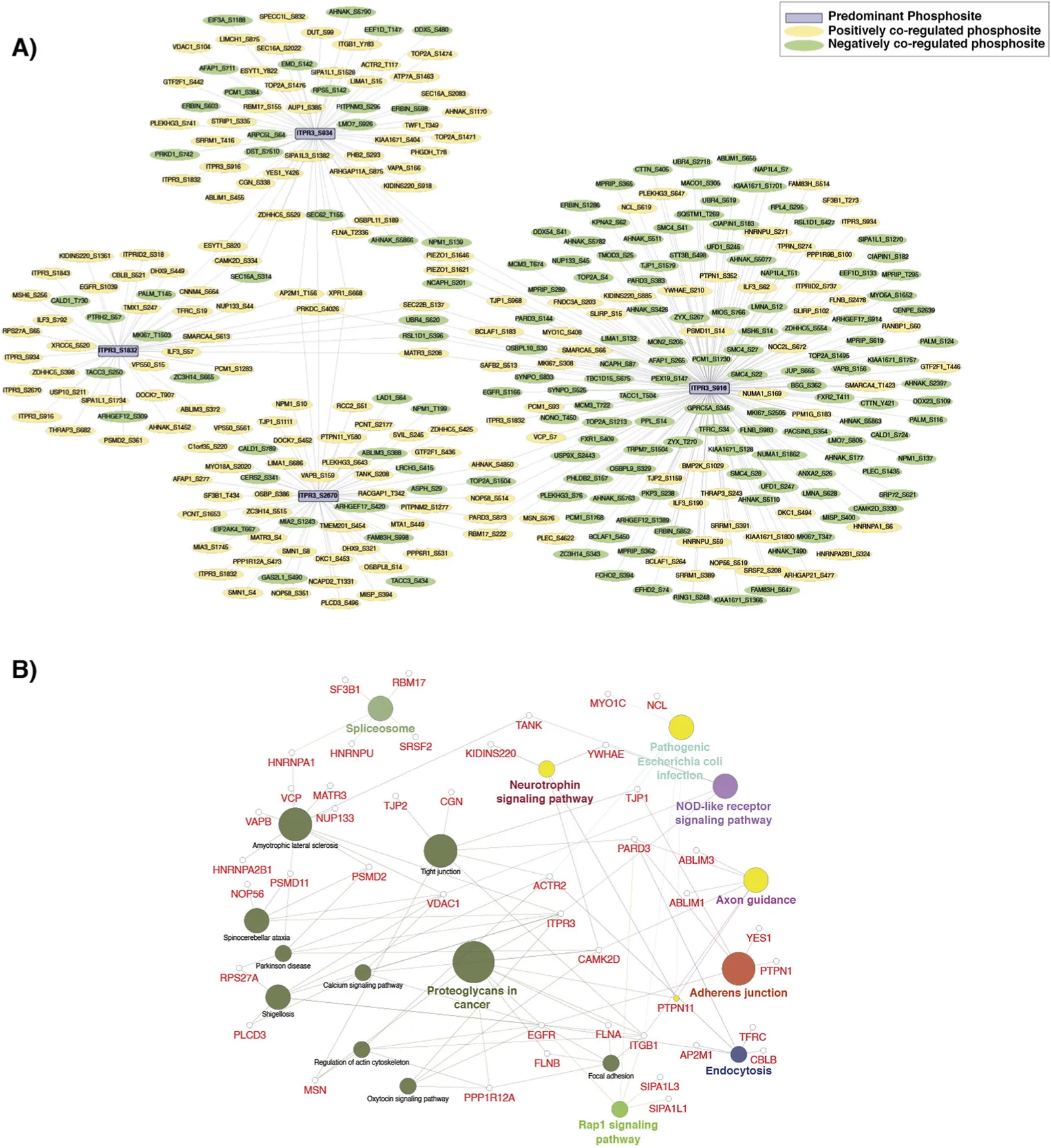
**(A)** Protein-protein interaction network of complex interactors associated with the predominant ITPR3 phosphosites (S916, S934, S1832, and S2670), showing positively and negatively co-regulated (PsOPs). Nodes represent complex-associated phosphosites identified among the co-regulated proteins. Yellow nodes indicate positively co-regulated phosphosites, whereas green nodes indicate negatively co-regulated phosphosites, and edges represent interactors. **(B)** ClueGO functional pathway enrichment analysis of the complex interactors associated with all four predominant ITPR3 phosphosites. Nodes represent significantly enriched pathways, grouped according to shared functional relationships. Node size corresponds to enrichment significance according to ClueGO (Bindea et al., 2009), while connecting edges indicate pathway interrelationships.

Further, functional enrichment analysis of proteins identified from the complex interaction networks was performed using ClueGO and CluePedia to investigate their associated biological pathways. The enriched network highlighted significant associations with proteoglycans in cancer, calcium signaling, Rap1 signaling, oxytocin signaling pathway, NOD-like receptor signaling pathway, focal adhesion, adherens junction, tight junction, endocytosis, axon guidance, and neurotrophin signaling pathways (Figure 8B).

The enrichment of the calcium signaling pathway is consistent with the established role of ITPR3 as a central regulator of intracellular Ca^2+^ homeostasis. Communication between the plasma membrane and the endoplasmic reticulum (ER), as well as between the ER and mitochondria, is essential for the regulation of intracellular Ca^2+^ transfer (Ambudkar et al., 2017). In this process, inositol 1,4,5-trisphosphate (IP_3_) acts as an important second messenger, that promotes Ca^2+^ release from intracellular stores by activating IP_3_ receptors (Putney, 1987; Park et al., 2020). Another notable finding was the enrichment of the Rap1 signaling pathway, which is closely associated with intracellular Ca^2+^ signaling. Previous studies have shown that Ca^2+^ release can activate Rap1, while Rap1 can also regulate Ca^2+^ signaling through several guanine nucleotide exchange factors (GEFs) (Kosuru and Chrzanowska, 2020). The enrichment of the proteoglycans in the cancer pathway is also noteworthy, as proteoglycans regulate cell-extracellular matrix (ECM) interactions and signaling networks involved in cancer progression, fibrosis, and tissue remodeling (Farach-Carson et al., 2024). Given the established involvement of Ca^2+^ signaling in cellular proliferation and disease progression (Rodrigues et al., 2007), the enrichment of this pathway may indicate a broader role for ITPR3-associated phospho-signaling in regulating cellular responses linked to tissue remodeling and cancer-associated signaling networks.

### 3.7 KEGG pathway analysis of ITPR3 phosphosite-associated co-regulated proteins

ITPR3 functions as an endoplasmic reticulum-localized Ca^2+^ release channel. By mediating the release of Ca^2+^ from intracellular stores, it plays a critical role in calcium homeostasis and intracellular signaling (Neumann et al., 2023; Zhang et al., 2025). Previous studies have shown that ITPR3 can influence Ca^2+^-dependent physiological processes by modulating intracellular calcium release (Soulsby and Wojcikiewicz, 2005). Therefore, to explore the potential functional significance of the predominant phosphosites identified in the present study, co-regulated proteins were mapped onto the KEGG calcium signaling pathway, the principal pathway in which ITPR3 functions.

Mapping the co-regulated proteins associated with each predominant ITPR3 phosphosite onto the KEGG calcium signaling pathway revealed that several co-regulated proteins were components of this pathway. These included Calcium/Calmodulin-Dependent Protein Kinase II Delta (CAMK2D, S334) and Epidermal Growth Factor Receptor (EGFR, S1039), which were positively co-regulated with the ITPR3 S1832 phosphosite. The positively co-regulated proteins of S934 predominant site were enriched in calcium signaling include Phosphorylase b kinase regulatory subunit beta (PHKB, S27; S700), Histamine Receptor H1 (HRH1, S380), CAMK2D (S334), Voltage-dependent anion channel 1 (VDAC1, S104), and Phosphorylase kinase regulatory subunit alpha 2 (PHKA2, S729). In contrast, Protein Tyrosine Kinase 2 Beta (PTK2B, Y576) was negatively co-regulated with the S934 predominant site. The calcium signaling-associated proteins detected among the ITPR3 S2670 (UUDD) group included Phospholipase C delta (PLCD3, S496), whereas Aspartate beta-hydroxylase (ASPH, S29) was identified in the UDDU. For S916 (UUDD), ITPR3, Histamine Receptor H1 (HRH1, S380), and PHKB (S27) were observed, while Stromal interaction molecule (STIM2, S697), PTK2B (S843), MET proto-oncogene, receptor tyrosine kinase (MET, T977; S45), EGFR (S1166), and CAMK2D (S330) were identified in the UDDU (Figure 9).

**FIGURE 9:**
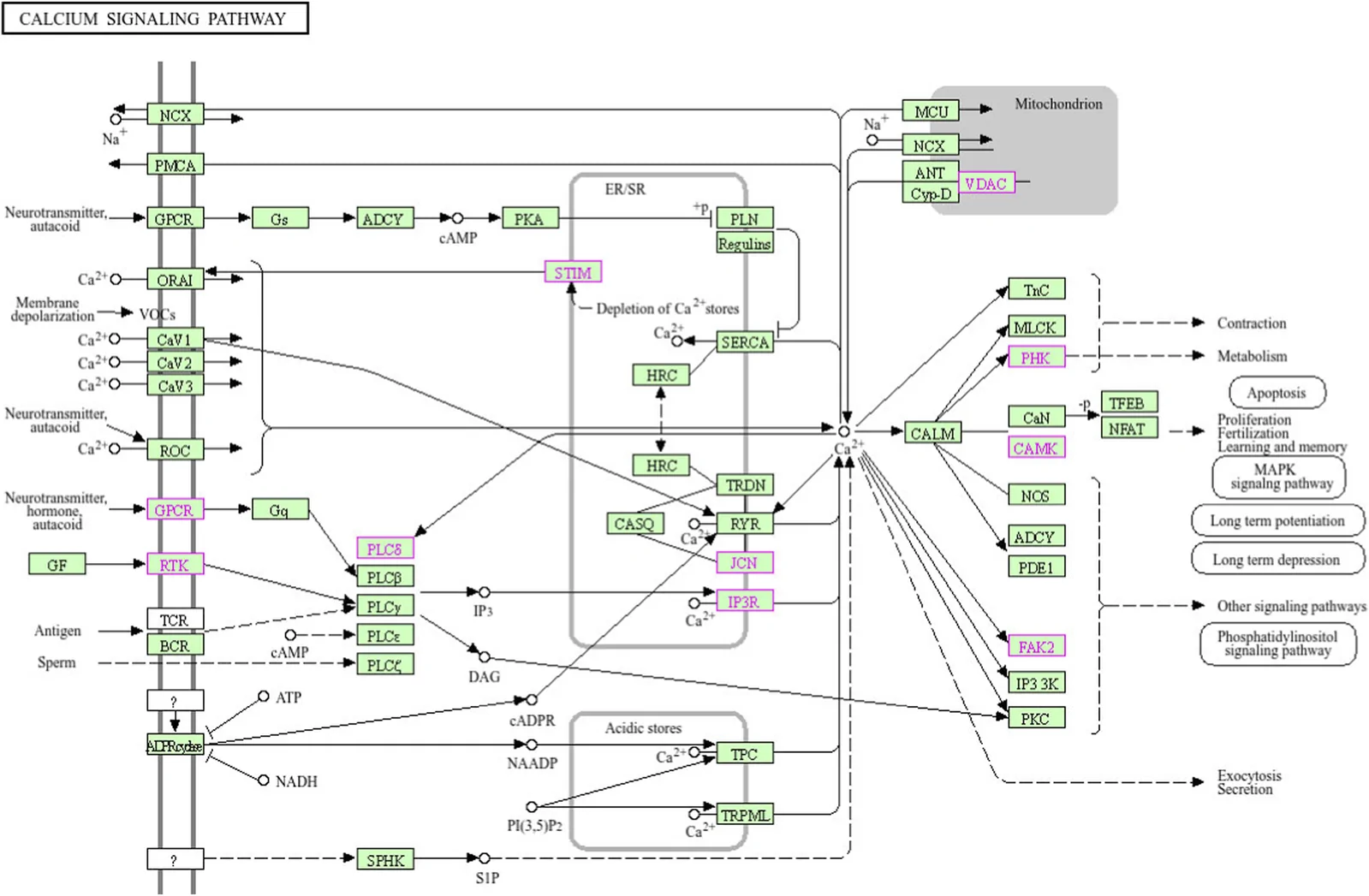
KEGG calcium signaling pathway showing proteins identified among the co-regulated proteins associated with the predominant ITPR3 phosphosites. Highlighted proteins represent components of the calcium signaling pathway.

These proteins occupy multiple functional modules of the calcium signaling pathway, including receptor-mediated signaling (EGFR) (Rodrigues et al., 2022), MET (Organ and Tsao, 2011; Maroun and Rowlands, 2014), HRH1 (Suzuki et al., 2018), IP3 generation (PLCD3) (Runkel et al., 2012), ER calcium release (ITPR3), store-operated calcium entry (STIM2) (Kodakandla et al., 2023), downstream calcium-responsive kinase signaling (CAMK2D and PTK2B), controls calcium ion flux into mitochondria (VDAC1) (Shoshan-Barmatz et al., 2018). Notably, ITPR3 has been reported to function as a critical Calcium transporter at mitochondria-associated membranes (MAMs), where it regulates ER-to-mitochondrial calcium transfer and intracellular calcium homeostasis (Zhu et al., 2025). The identification of VDAC1 among the proteins co-regulated with the predominant ITPR3 phosphosites suggests that phosphorylation-dependent regulation of ITPR3 may influence broader calcium signaling processes associated with ER-mitochondrial communication. The enrichment of calcium signaling pathway components among proteins co-regulated with the predominant ITPR3 phosphosites is consistent with the established role of ITPR3 as a major endoplasmic reticulum Ca^2+^ release channel. Upon activation of GPCR and receptor tyrosine kinase-mediated signaling pathways, phospholipase C generates IP_3_, which binds to IP3 receptors and triggers Ca^2+^ release from intracellular stores (Werry et al., 2003). The identification of proteins involved in receptor signaling, IP_3_ generation (PLCD3), calcium-responsive signaling, and mitochondrial calcium handling may suggest that phosphorylation of the predominant ITPR3 sites may be associated with distinct regulatory modules of the calcium signaling cascade.

Numerous signaling pathways converge on IP_3_-mediated Ca^2+^ signaling, underscoring its importance as a central signaling hub (Shibao et al., 2010). Given the established roles of Ca^2+^ signaling in regulating apoptosis and tumor growth (Mendes et al., 2005; Rodrigues et al., 2007), the identification of multiple calcium signaling components within the co-regulated networks of the predominant ITPR3 phosphosites suggests that these phosphorylation events may contribute to broader signaling mechanisms linked to cellular growth and survival.

### 3.8 Analysis of predominant ITPR3 phosphosites in cancer cell lines

ITPR3 expression has been associated with the development of several human malignancies, including glioblastoma, breast cancer, gastric cancer, and head and neck squamous cell carcinoma (Mangla et al., 2020). As an important regulator of intracellular Ca^2+^ signaling, ITPR3 participates in cellular processes that are relevant to cancer development and progression. Cancer cells can alter the expression and activity of IP3 receptors by modulating various proto-oncogenes and tumor suppressor genes, thereby promoting cell survival and malignant transformation (Akl and Bultynck, 2013). In addition, ITPR3 gene mutations have been detected in metastatic or recurrent tumors but not in their corresponding primary tumors, suggesting that ITPR3 may be involved in cancer progression. Supporting this observation, silencing of ITPR3 significantly impairs tumor growth in both *in vitro* and *in vivo* settings, providing direct evidence of its contribution to carcinogenesis (Gambardella et al., 2020).

To gain insight into the phosphorylation-dependent regulation of ITPR3, we analyzed curated differential phosphoproteomic datasets obtained from a range of human cell types, including fibroblasts, cancer-derived cell lines, and transformed cell lines. As this study aimed to investigate the role of ITPR3 phosphorylation in cancer, further analyses were restricted to datasets derived from cancer cell lines. Among the predominant phosphosites, S1832 was the most frequently observed, appearing in 82 of 98 datasets from cancer cell lines. Similarly, S934 was identified in 65 of 76 cancer-derived datasets, S2670 in 55 of 63 datasets, and S916 in 17 of 24 datasets. These cancer-associated datasets were categorized by their phosphorylation status (upregulated or downregulated) for further analysis. Cancer-specific distribution of ITPR3 phosphosites across differential datasets was provided in Supplementary Table S14.

S1832 showed the highest detection frequency across cancer cell lines, particularly in skin cancer, followed by blood, cervical, and lung cancers. Phosphorylation of S1832 was generally upregulated in skin, blood and cervical cancers, whereas lung cancer datasets showed downregulation. S934 was also widely distributed across multiple cancer types with the highest occurrence in lung, skin, colon and cervical cancers. S934 was mainly downregulated in lung and liver cancers it was largely upregulated in skin, colon, breast, cervical and blood cancers. S2670 was detected mainly in breast and lung cancers, followed by cervical, skin, blood, colon and liver cancers. In contrast, S916 was identified in comparatively fewer cancer datasets and found to be upregulated in cervical, lung, skin, colon, blood and bone cancers (Figure 10).

**FIGURE 10:**
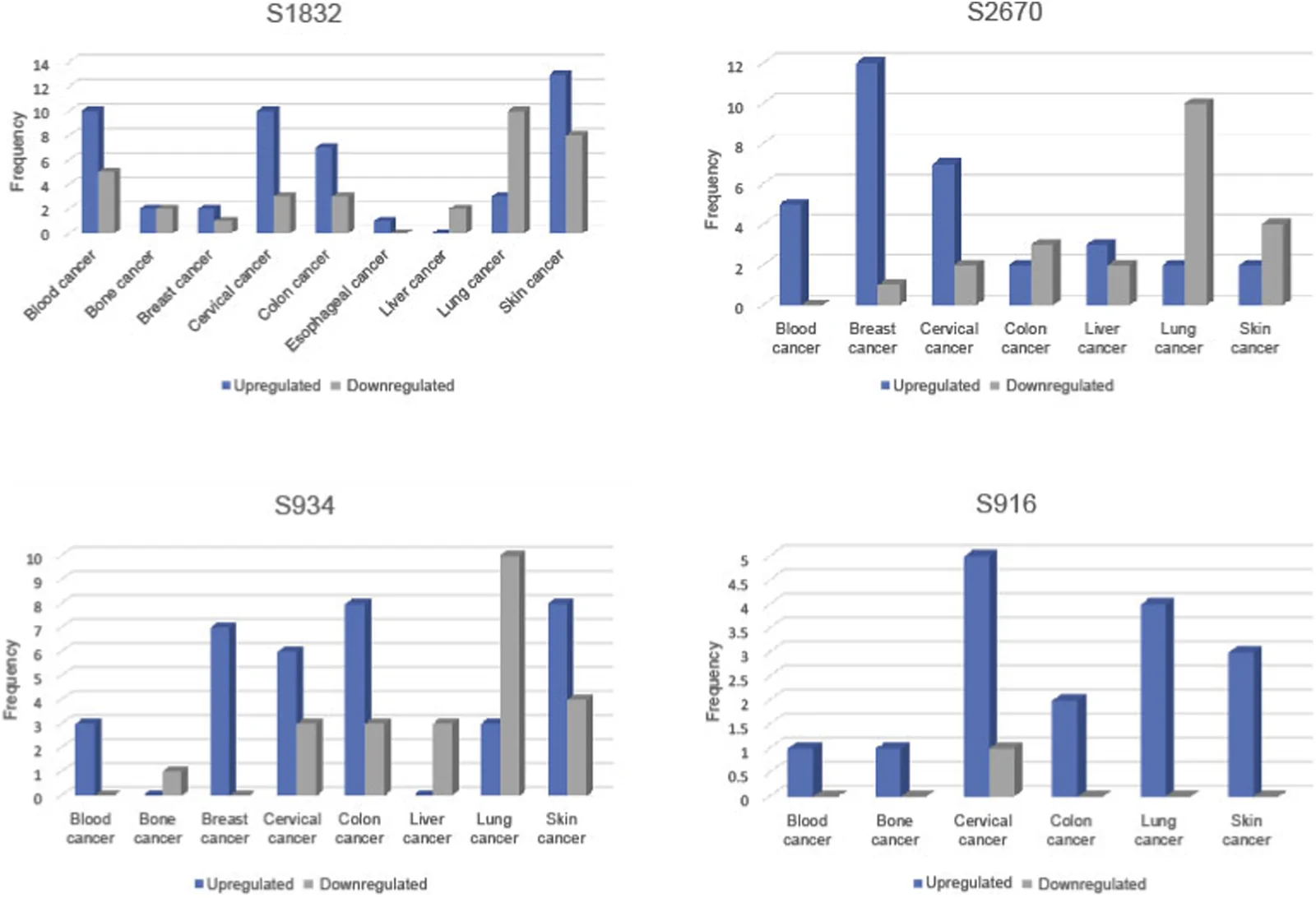
Bar plot depicting the site-specific phosphorylation pattern of ITPR3 across distinct cancers. The y-axis depicts the frequency of upregulated and downregulated phosphorylation events for four predominant phosphosites. The x-axis represents the cancer types.

### 3.9 Analysis of ITPR3 phosphorylation across human cancers using cProSite

To further assess the cancer-associated phosphorylation profile of ITPR3, phosphosite-level data were analyzed using cProSite. Phosphorylation changes were evaluated based on log_2_ fold-change (log_2_FC) values. Among the detected ITPR3 phosphosites, the four predominant sites such as S1832, S934, S2670, and S916, showed the distribution across multiple tumor types. In contrast, most other phosphosites occurred less frequently and were detected in a limited number of cancer types. Notably, all four predominant phosphosites showed variation in log_2_FC values across different tumor types, indicating that their phosphorylation levels varied across cancer types. (Figure 11A).

**FIGURE 11:**
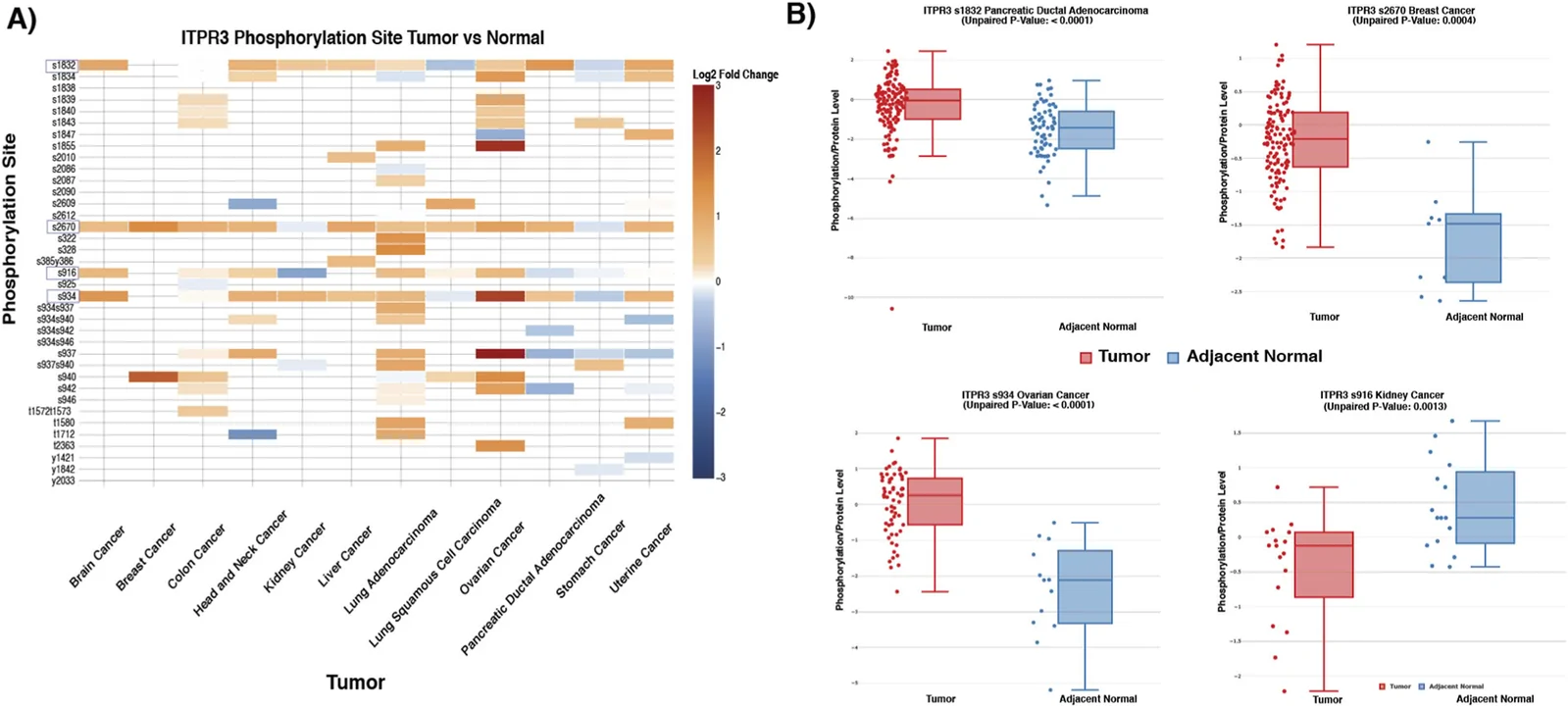
**(A)** Heatmap showing differential phosphorylation of ITPR3 phosphosites across multiple cancer types based on cProSite data. Color intensity represents log_2_ fold-change relative to normal tissues. **(B)** Tumor *versus* adjacent normal phosphorylation levels of the predominant ITPR3 phosphosites. Corresponding p-values are indicated in each panel.

For each predominant phosphosite, the cancer type exhibiting the most significant differential phosphorylation was selected for analysis. S1832 showed significant upregulation in pancreatic ductal adenocarcinoma (log_2_FC = 1.2909, p-value <0.0001), while S2670 was significantly upregulated in breast cancer (log_2_FC = 1.4838, p-value = 0.0004). Among the four predominant phosphosites, S934 exhibited the largest increase in phosphorylation in ovarian cancer, with a log_2_FC of 2.4224 (p < 0.0001). In contrast, S916 was significantly downregulated in kidney cancer (log_2_FC = −0.8818, p-value = 0.0013) (Figure 10B). These findings provide exploratory evidence of cancer type-specific variation in ITPR3 phosphosite phosphorylation and generate hypotheses regarding potential site- and cancer-specific regulatory patterns that warrant further investigation. The complete cProSite dataset of all four predominant phosphosites, including fold changes, p-value and sample sizes for all analyzed tumor types, is provided in Supplementary Table S15.

Previous studies have highlighted diverse roles of ITPR3 in cancer biology. As a key regulator of intracellular Ca^2+^ signaling, ITPR3 promotes breast cancer cell migration by modulating cytoskeletal organization (Vautrin-Glabik et al., 2018). In addition, increased ITPR3 expression contributes to estrogen-driven proliferation and altered Ca^2+^ signaling in breast cancer, supporting a role in tumor growth and progression (Szatkowski et al., 2010). Phosphorylation-dependent regulation of ITPR3 has also been shown to alter Ca^2+^ signaling in pancreatic acinar cells (Straub et al., 2002). Although these studies establish a role for ITPR3 in calcium-associated signaling and cancer-related processes, direct evidence linking the predominant ITPR3 phosphosites identified in this study to the regulation of calcium signaling is currently limited. These phosphosites may therefore contribute to the modulation of ITPR3-associated calcium signaling or downstream cellular responses rather than directly controlling Ca^2+^ channel gating. However, their specific functional effects require experimental validation. Beyond its role in Ca^2+^ signaling, ITPR3 contributes to cancer cell survival, as overexpression of ITPR3 suppresses apoptosis in breast and colorectal cancer cells, whereas knockdown or silencing promotes apoptotic cell death in breast, colorectal, and renal cancer cells (Rezuchova et al., 2019; Wu et al., 2021). Increased ITPR3 expression has also been reported in colorectal cancer (Shibao et al., 2010). Similarly, ITPR3 contributes to hepatocarcinogenesis by promoting resistance to apoptosis and supporting tumor cell survival (Dos Santos et al., 2022). Furthermore, ITPR3 contributes to the peritoneal spread of gastric cancer and may therefore serve as a promising molecular target for therapeutic intervention (Sakakura et al., 2003).

## 4 Conclusion

This study provides a detailed analysis of ITPR3 phosphorylation and regulatory networks associated with its predominant phosphosites S1832, S934, S2670 and S916. Integration of large-scale phosphoproteomics datasets enabled the identification of recurrent phosphorylation events, co-regulated PsOPs, potential upstream kinases, and protein interaction networks associated with these predominant sites. The co-regulation patterns observed for each phosphosite suggest that ITPR3 phosphorylation is highly site-specific and may participate in diverse cellular processes rather than functioning through a uniform regulatory mechanism. Functional enrichment analyses revealed associations with chromatin organization, transcriptional regulation, RNA processing, cytoskeletal dynamics, and signaling pathways, indicating that individual ITPR3 phosphosites may contribute to different biological functions. In addition, mapping of co-regulated proteins onto the calcium signaling pathway identified several pathway-associated components supporting the central role of ITPR3 phosphorylation in regulating intracellular Ca^2+^ signaling networks.

Our analysis also demonstrated enrichment of the predominant ITPR3 phosphosites in cancer-associated phosphoproteomics datasets, with a high frequency of occurrence across diverse cancer cell lines. Analysis of the cell-of-origin of cancer, revealed that all four predominant sites were widely represented across multiple tumor types, while cProSite tumor phosphoproteomic data demonstrated distinct tumor-specific phosphorylation patterns. Together, these findings suggest that phosphorylation-dependent regulation of ITPR3 may be associated with cancer-specific signaling programs. Given the established role of ITPR3-mediated Ca^2+^ signaling in cell proliferation, apoptosis, migration, and tumor progression, the recurrent detection of these phosphosites in cancer-related datasets suggests that they may be relevant to ITPR3 regulation in cancer. In addition, the identification of candidate upstream kinases and interaction networks of co-regulated proteins provides a framework for understanding the phosphoregulatory landscape of ITPR3. Together, these findings expand the current knowledge of ITPR3 phosphorylation in the context of calcium signaling and cancer.

## 5 Limitations

Although this study provides a valuable resource of the site-specific phosphoregulatory network of ITPR3, certain limitations should be considered. As this study integrates multiple large-scale phosphoproteomic datasets, it may introduce inter-study variability in experimental design, sample types*,* mass spectrometry platforms, and data processing methods. The use of publicly available mass spectrometry data presents inherent variability from differences in experimental conditions, sample types and data quality across studies. Data normalization and batch-effect correction across all datasets could not be performed due to limited access to raw data and the lack of a unified data reprocessing workflow. In addition, the curated datasets included both label-based (e.g., TMT and SILAC) and label-free quantification approaches. Although fold-change thresholds were applied within each individual quantitative dataset and were not directly compared across different quantification strategies, differences in quantitative dynamic range between these approaches may still contribute to variability in the observed fold-change patterns. The protein mapping was based on the May 2023 UniProt release; therefore, subsequent updates to protein annotations may not be captured in the present analysis. However, to reduce potential biases, stringent filtering criteria, based on Fisher’s Exact Test significance, experimental confidence scores, and PMID confidence, were applied to identify high-confidence phosphosites in co-regulated proteins. The co-regulatory relationships, protein interaction networks, pathway associations, and kinase-substrate interactions identified in this study are based on computational and bioinformatics analyses and therefore require further experimental validation. In particular, the functional significance of the predominant ITPR3 phosphosites and their proposed roles in calcium signaling and cancer-associated pathways should be validated by using phosphosite-directed mutagenesis, kinase assays, and cellular functional studies. Nevertheless, this study provides a valuable framework for future investigations into the phosphoregulatory mechanisms of ITPR3 and their potential roles in human disease.

## Data Availability

The original contributions presented in the study are included in the article/Supplementary Material, further inquiries can be directed to the corresponding authors.

## Supplementary material

SUPPLEMENTARY FIGURE S1 Multiple sequence alignment of ITPR1, ITPR2, and ITPR3 performed using Clustal Omega.

SUPPLEMENTARY FIGURE S2 Top 10 hub genes identified using the CytoHubba plugin based on network connectivity.
